# Supplementary material for: Structural insights into the mechanism and inhibition of transglutaminase-induced ubiquitination by the Legionella effector MavC
Source: Nat Commun. 2020 Apr 14;11:1774. doi: 10.1038/s41467-020-15645-7 (PMC7156659; doi:10.1038/s41467-020-15645-7)
Supplement: Supplementary file 1 — Supplementary Information [file 41467_2020_15645_MOESM1_ESM.pdf]

Supplementary Information

Structural insights into the mechanism and inhibition of transglutaminase-induced ubiquitination by the *Legionella* effector MavC

Yajuan Mu, Yue Wang, Yanfei Huang, Dong Li, Youyou Han, Min Chang, Jiaqi Fu, Yongchao Xie, Jie Ren, Hao Wang, Yi Zhang, Zhao-Qing Luo, Yue Feng

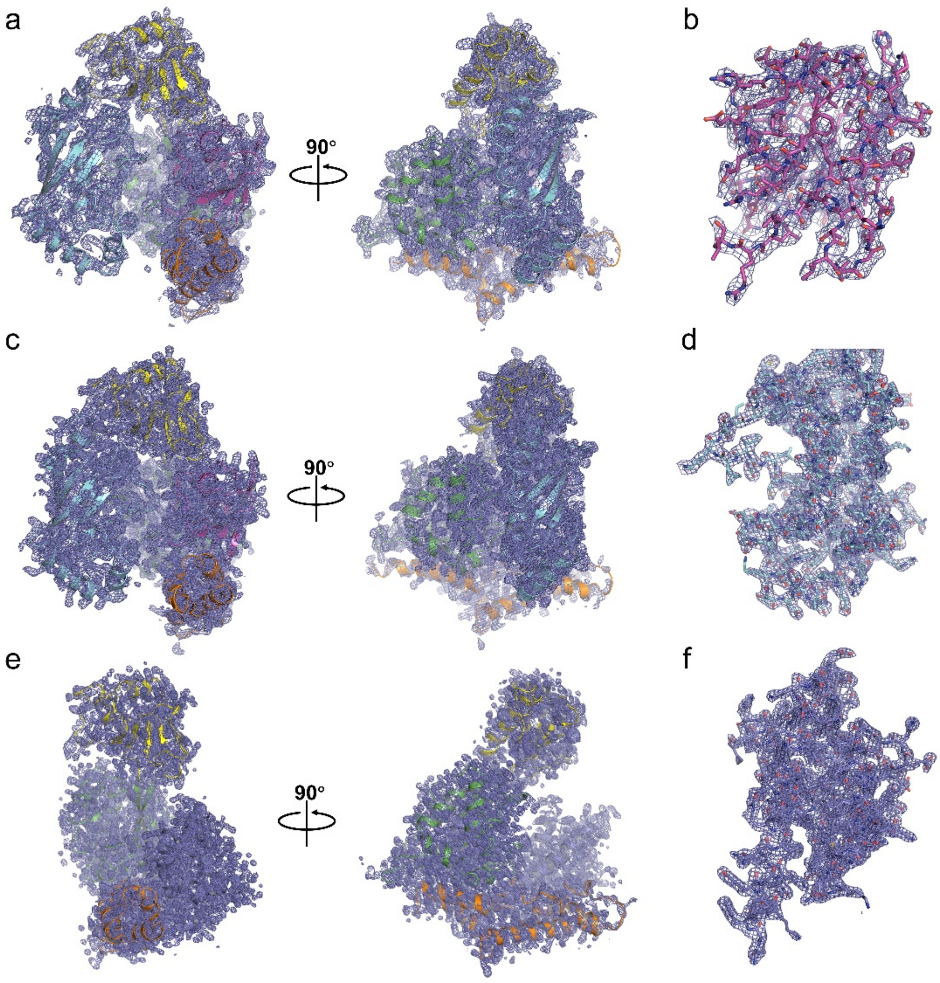

Supplementary Fig. 1 Electron density of the structures reported in this study

a-b. The 2Fo-Fc electron density maps contoured at 1σ are shown in blue mesh for the overall structure of MavC/UBE2N/Ub ternary complex (a) and the Ub molecule (b).

c-d. The 2Fo-Fc electron density maps contoured at 1σ are shown in blue mesh for the overall structure of MavC/UBE2N-Ub binary complex (c) and a part of the UBE2N molecule (d).

e-f. The 2Fo-Fc electron density maps contoured at 1σ are shown in blue mesh for the overall structure of MavC/Lpg2149 binary complex (e) and the Lpg2149 molecule (f).

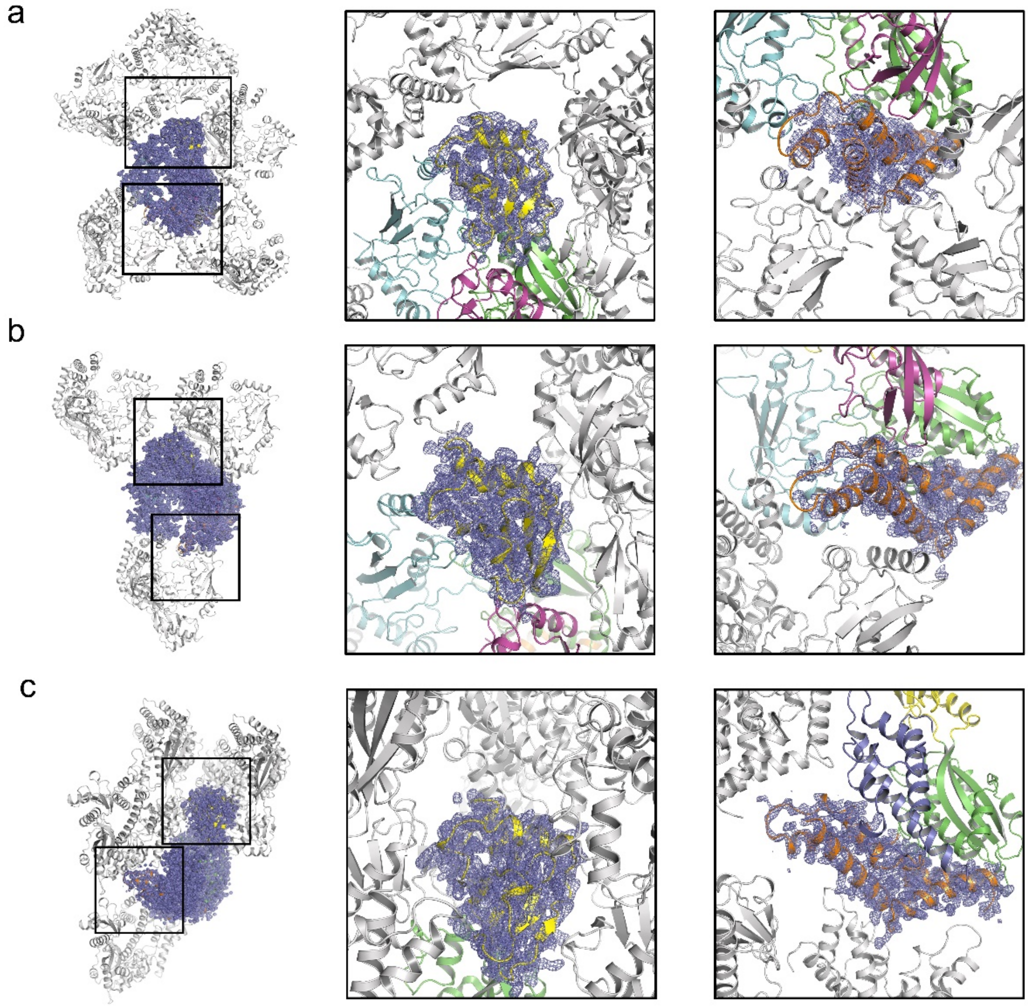

Supplementary Fig. 2 Crystal packings around the insertion domain and the helical-bundle region of MavC in the structures reported in this study

a-c. Crystal packings around the insertion domain and the helical-bundle region of MavC are shown in the MavC/UBE2N/Ub ternary complex (a), the MavC/UBE2N-Ub binary complex (b) and the MavC/Lpg2149 binary complex (c). The central complexes are colored as in Fig. 1a (a and b) and Fig. 4a (c) with the 2Fo-Fc electron density map contoured at 1σ shown in blue mesh, and the symmetry molecules are colored in grey. Enlarged views of the insertion domain and the helical-bundle region of MavC for each structure are shown in the middle and right in each panel, with the electron density only shown for the insertion domain (middle) and helical-bundle region of MavC (right). The results showed that crystal packing might affect the conformational change between the helical bundle regions of MavC in the MavC/UBE2N-Ub and

MavC/Lpg2149 complexes, but may exert little effect in conformational change between the insertion domains of MavC in the MavC/UBE2N/Ub complex and apo MavC.

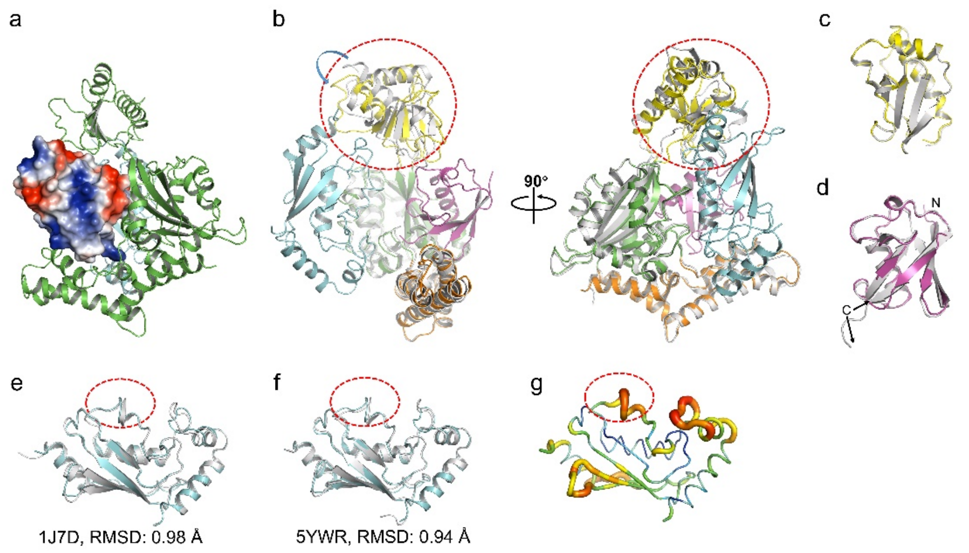

Supplementary Fig. 3 Structural superimposition between the MavC/UBE2N/Ub complex and respective apo proteins.

a. Another view of Fig. 1d by a clockwise rotation of 130°.

b. Structural superimposition between MavC/UBE2N/Ub and apo MavC (PDB code: 5TSC). The MavC/UBE2N/Ub complex is colored as in Fig. 1a and apo MavC is colored grey. Two views are shown. The arrow indicates the orientation of rotation.

c. Structural superimposition between the insertion domains of MavC in the MavC/UBE2N/Ub complex and apo MavC, colored the same as in a.

d. Structural superimposition between Ub of the MavC/UBE2N/Ub complex and apo Ub (PDB code: 1UBQ). Ub of the MavC/UBE2N/Ub complex is colored magenta, and the apo Ub is in gray.

e-f. Structural superimposition between UBE2N of the MavC/UBE2N/Ub complex and two available UBE2N structures. The PDB codes and RMSD values are shown below each superimposition figure. UBE2N of the MavC/UBE2N/Ub complex is colored cyan, and the other structures are colored grey in each figure. The K92/K94 loop region is marked in a circle.

g. Analysis of intramolecular B-factor values in UBE2N in the structure of

MavC/UBE2N/Ub complex. UBE2N is shown in cartoon, with blue representing the lowest and red the highest B-factor values. The size of the tube also reflects the B-factor values: the higher the B-factor, the thicker the tube. The K92/K94 loop region is marked in a circle.

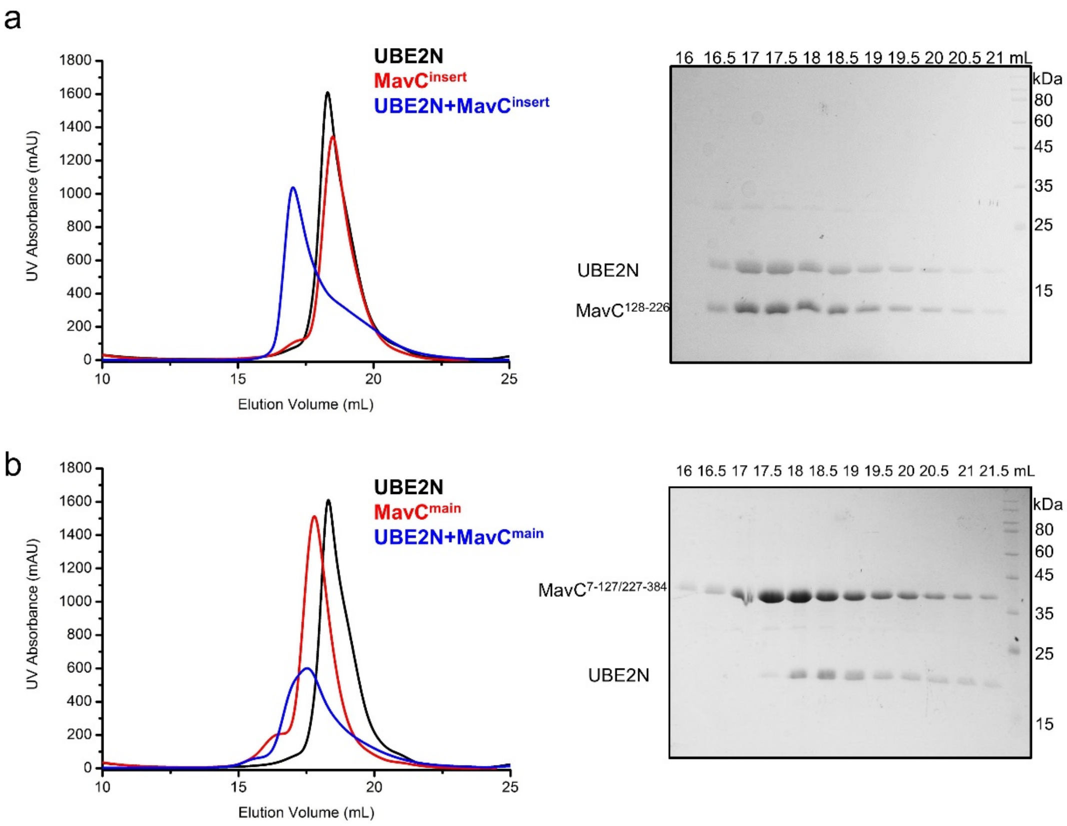

Supplementary Fig. 4 Gel filtration assay of UBE2N and two domains of MavC

a. The gel filtration profiles of MavC^{insert}, UBE2N and the mixture of them are shown. The fractions were subjected to SDS-PAGE gel and shown on the right.

b. The gel filtration profiles of MavC^{main}, UBE2N and the mixture of them are shown. The fractions were subjected to SDS-PAGE gel and shown on the right. Source data are provided as a Source Data file. Experiments in a and b were repeated independently three times with similar results.

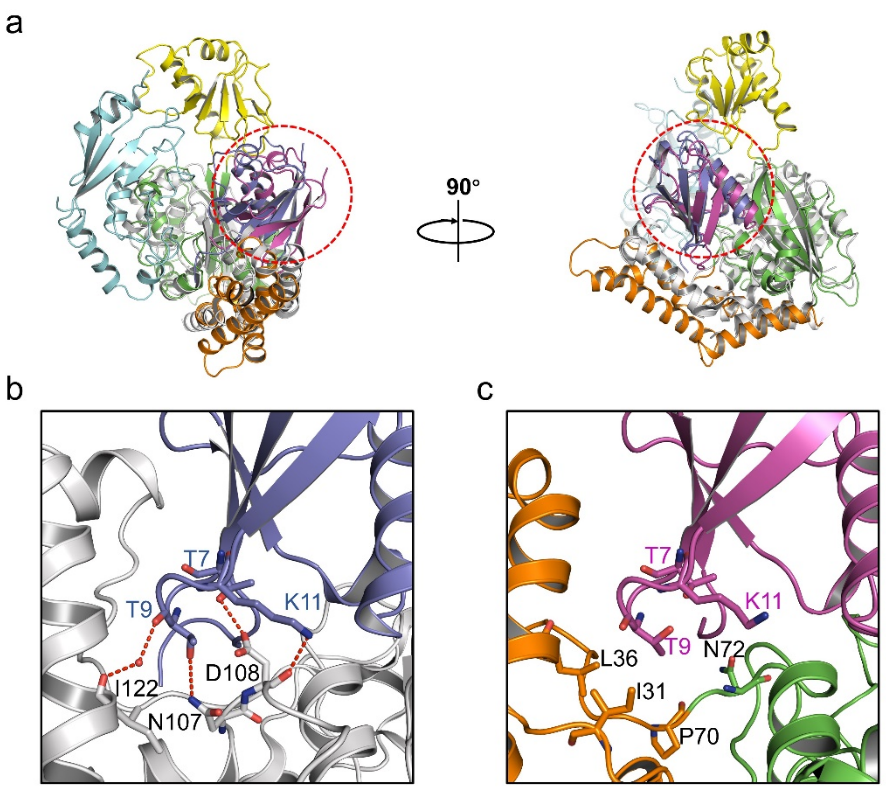

Supplementary Fig. 5 Structural alignment between the MavC/UBE2N/Ub complex and CHBP/Ub complex

a. Structural alignment between the MavC/UBE2N/Ub complex and CHBP/Ub complex (PDB code: 4HCN). The MavC/UBE2N/Ub complex is colored as in Fig. 1a. CHBP and Ub in the CHBP/Ub complex are colored grey and blue, respectively.

b-c. The Ub^{K11} regions of the two structures in a are shown in details. All the molecules are colored as in a. Hydrogen bonds are shown as red dashed lines.

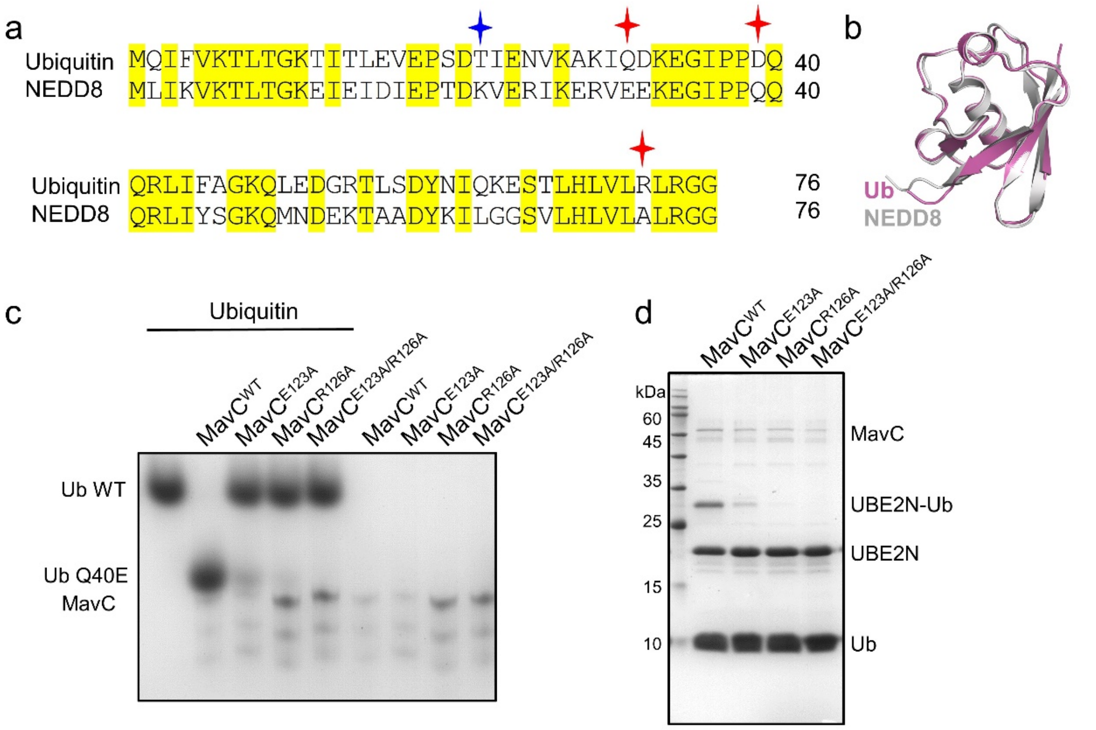

Supplementary Fig. 6 Recognition of Ub by MavC

a. Sequence alignment between human ubiquitin and NEDD8. The Ub residues involved in MavC binding and different from those of NEDD8 are marked with red stars. Ub^{T22} and $NEDD8^{K22}$ is marked with a blue star.

b. Structural alignment between Ub in the MavC/UBE2N/Ub complex and NEDD8.

c. MavC and its mutants were incubated with Ub or not, for 2 hours at 37 ℃. Then the samples were subjected to native gel, followed by Coomassie blue staining. Source data are provided as a Source Data file.

d. MavC and its mutants were incubated with UBE2N and Ub for 1 hour at 37 ℃. Then the samples were subjected to Tricine gel, followed by Coomassie blue staining. Source data are provided as a Source Data file. Experiments in c and d were repeated independently three times with similar results.

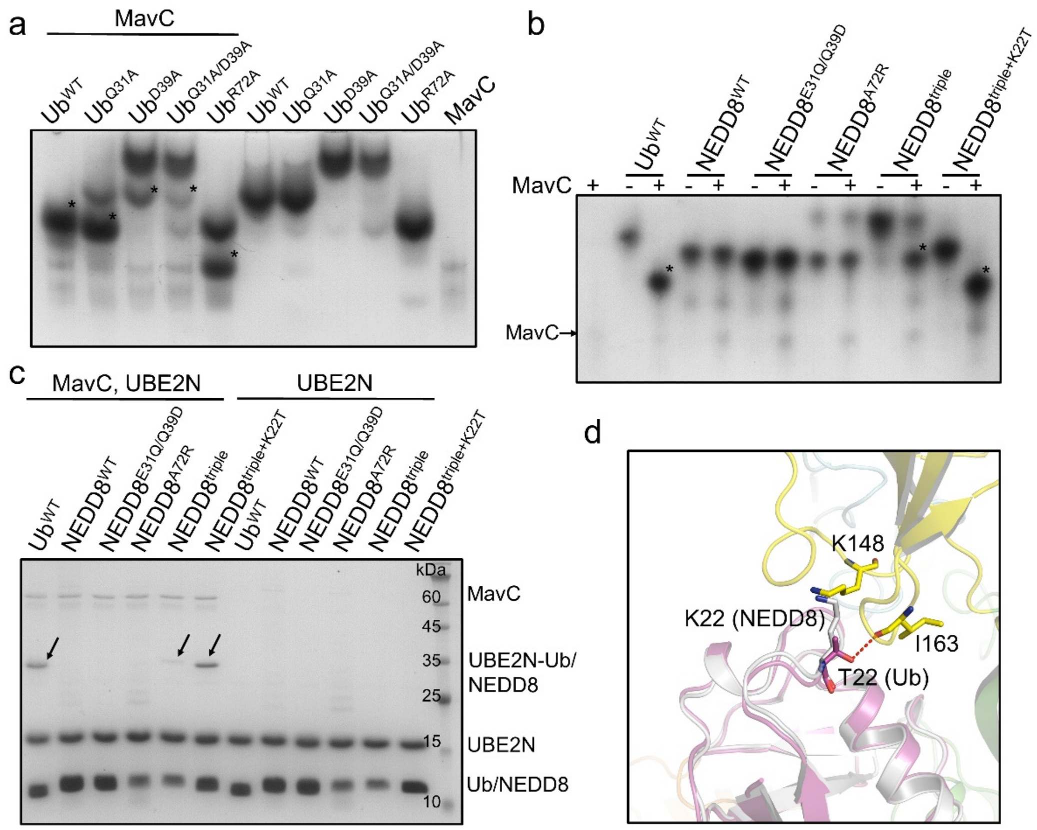

Supplementary Fig. 7 Substrate specificity of MavC

a. Ub and its mutants were incubated with MavC or not, for 2 hours at 37 ℃. Then the samples were subjected to native gel, followed by Coomassie blue staining. The deamidated species are labeled with asterisks. Source data are provided as a Source Data file.

b. Ub, NEDD8 and its mutants were incubated with MavC or not, for 3 hours at 37 ℃. Then the samples were subjected to native gel, followed by Coomassie blue staining. NEDD8^{triple} indicates the NEDD8$^{E31Q/Q39D/A72R}$ mutant. The deamidated species are labeled with asterisks. Source data are provided as a Source Data file.

c. Ub, NEDD8 and its mutants were incubated with MavC and UBE2N, or UBE2N itself, for 1 hour at 37 ℃. Then the samples were subjected to Tricine gel, followed by Coomassie blue taining. The ubiquitination products are marked with arrows. Source data are provided as a Source Data file. Experiments in a-c were repeated independently three times with similar results.

d. Structural superimposition of NEDD8 and the MavC/UBE2N/Ub complex. The T22 of Ub (K22 in NEDD8) region is shown in detail. MavC and Ub are colored in green

and magenta, respectively. NEDD8 is shown in yellow. The red dashed line represents a hydrogen bond.

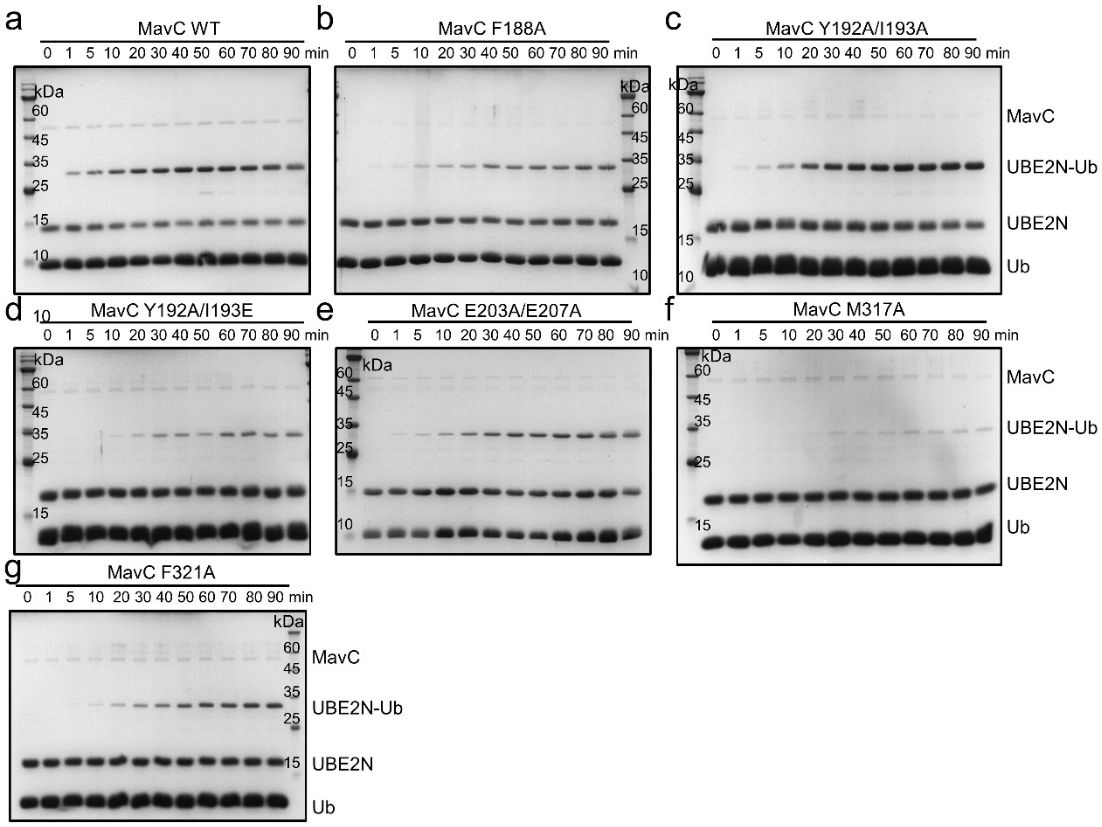

Supplementary Fig. 8 Kinetic studies of MavC and its mutants related to Fig. 2g a-g. MavC and indicated mutants were incubated with UBE2N and Ub at 37 °C for the indicated amounts of time. The samples were analyzed using Coomassie blue staining. The quantification results are shown in Fig. 2g. Source data are provided as a Source Data file. Experiments in a-g were repeated independently three times with similar results.

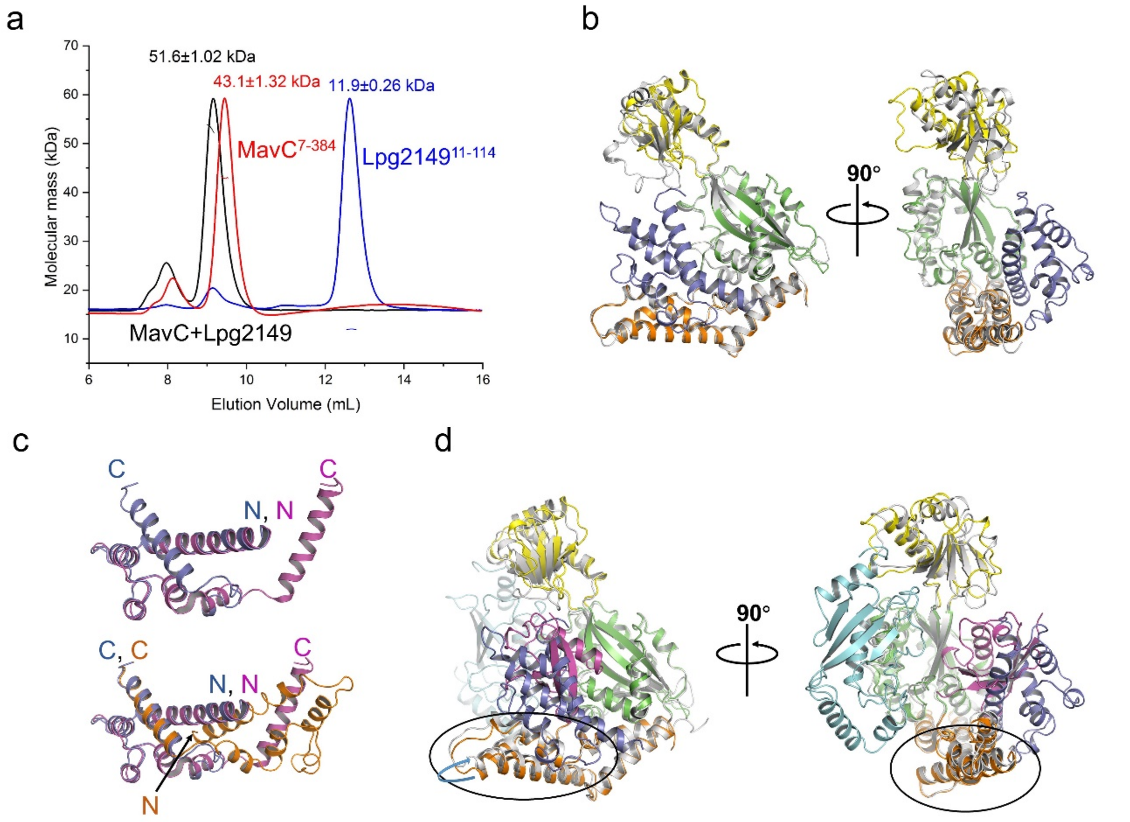

Supplementary Fig. 9 Structure of the MavC/Lpg2149 complex

a. Static light scattering (SLS) studies of MavC$^{7\text{-}384}$, Lpg2149$^{11\text{-}114}$ and the mixture of them. The calculated molecular weights of the main peaks of the three profiles are shown above the peaks.

b. Structural superimposition between the MavC/Lpg2149 complex and apo MavC (PDB code: 5TSC).

c. Structural superimposition between Lpg2149 (blue) in the MavC/Lpg2149 complex and one protomer (magenta) in the study of Valleu et al (PDB code: 5DPO, upper panel), and between Lpg2149 (blue) in the MavC/Lpg2149 complex and the dimer form (magenta and orange) of PDB 5DPO (lower panel).

d. Structural superimposition between the MavC/Lpg2149 complex and the MavC/UBE2N-Ub complex. The MavC/UBE2N-Ub complex is colored as in Fig. 1a. MavC and Lpg2149 in the MavC/Lpg2149 complex are colored grey and blue, respectively. Marked in the circle is the helical-bundle tail region of MavC. The arrow indicates the orientation of rotation.

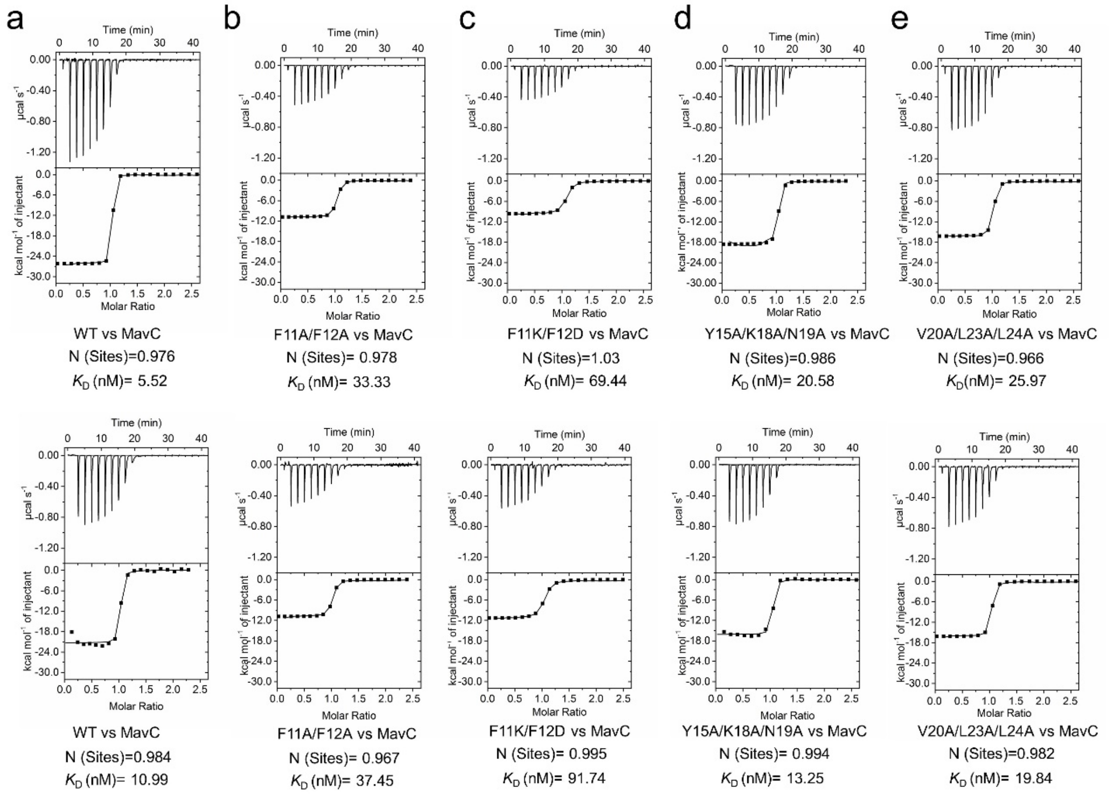

Supplementary Fig. 10 ITC titration experiments of Lpg2149 and its mutants binding with MavC

a-e. ITC assays to test binding of Lpg2149 and its mutants to MavC are shown. All titrations were performed at 25 ℃ in 20 mM HEPES pH 7.5, 200 mM NaCl. The K_D and N parameters obtained from the fits are shown as text below the raw data.

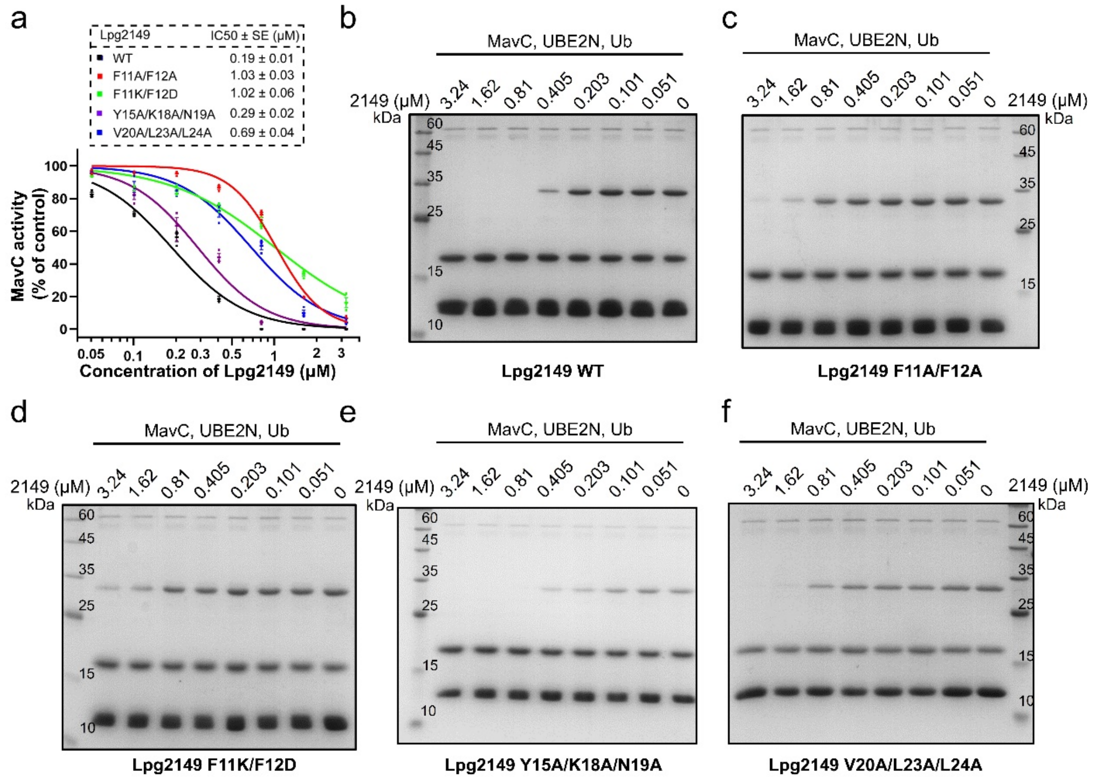

Supplementary Fig. 11 IC50 analysis of Lpg2149 and its mutants

a. Inhibition of MavC by Lpg2149 and its mutants. The activity of MavC in the presence of Lpg2149 or its mutants was obtained by the band intensities of UBE2N-Ub on the gel quantified by ImageJ. The amount of UBE2N-Ub generated by the reaction with Lpg2149 of indicated concentrations was then normalized to the amount generated by MavC with no Lpg2149 on each gel. The data were fitted and IC50 values were calculated by the GraphPad Prism 8.0.1 according to the dose-response model with variable slope. Each experiment was repeated three times and mean ± s.e.m. is shown for each data point. Source data are provided as a Source Data file.

b-f. Representative gels for the results shown in a. The experiments were performed as stated in the Methods section. Source data are provided as a Source Data file. Experiments in b-f were repeated independently three times with similar results.

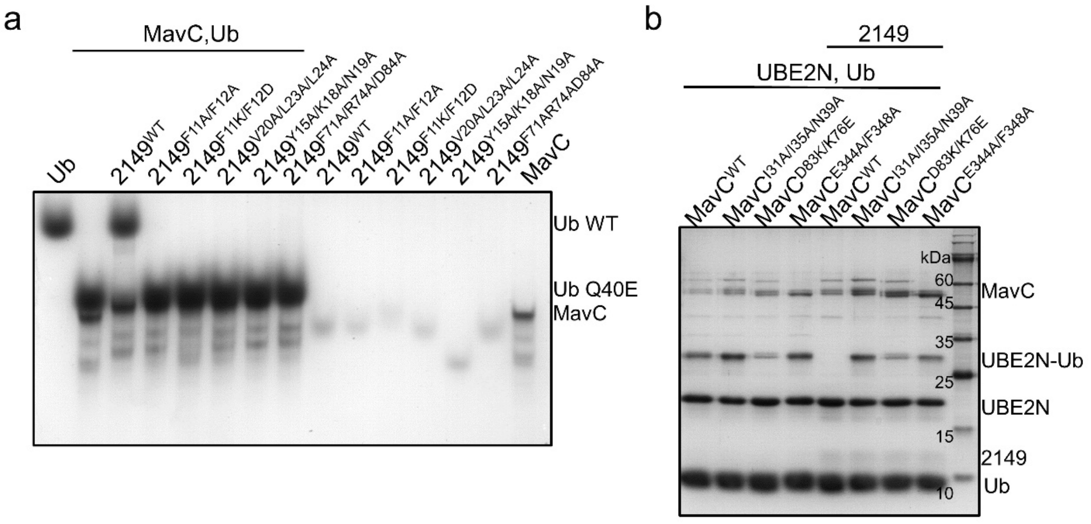

Supplementary Fig. 12 Activity assays to test the binding between MavC and Lpg2149

a. Lpg2149 and it mutants were incubated with MavC and Ub or not, for 3 hours at 37 ℃. Then the samples were subjected to native gel, followed by Coomassie blue staining. Source data are provided as a Source Data file.

b. MavC and it mutants were incubated with UBE2N and Ub, in the presence of Lpg2149 or not, for 2 hours at 37 ℃. Then the samples were subjected to Tricine gel, followed by Coomassie blue staining. Source data are provided as a Source Data file. Experiments in a and b were repeated independently three times with similar results.

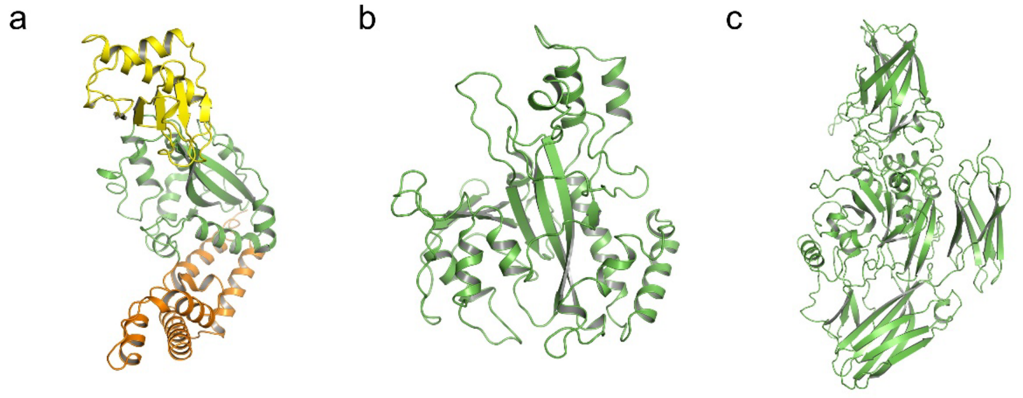

Supplementary Fig. 13 MavC is a novel family of transglutaminase

a-c. Structures of MavC (a), the transglutaminase from *Streptoverticillium mobaraense* (PDB code: 1IU4) (b) and human blood coagulation factor XIII (PDB code: 1GGT) (c).

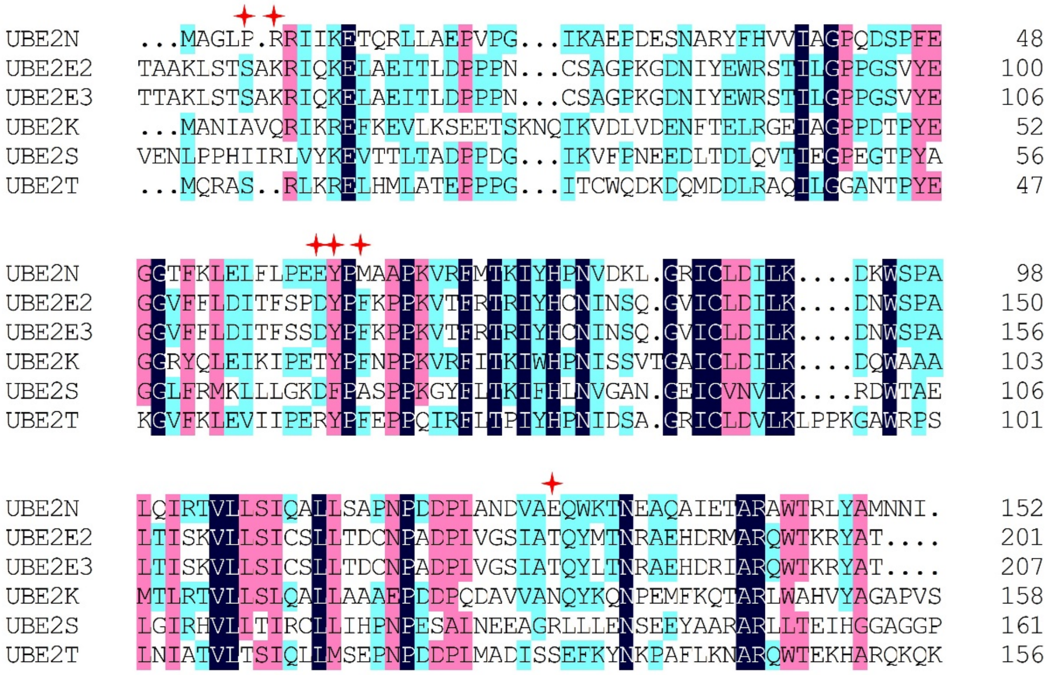

Supplementary Fig. 14 Sequence alignments of UBE2N homologs

Sequence alignment among UBE2N homologs from *Homo sapiens*. The residues involved in MavC binding are indicated.

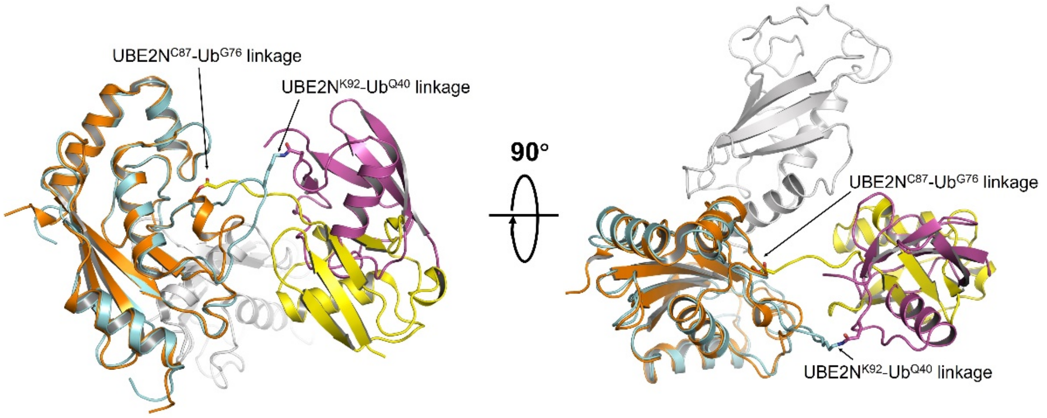

Supplementary Fig. 15 The mechanism of activity inhibition of UBE2N by MavC-catalyzed ubiquitination

Structural superimposition between the UBE2N-Ub product by MavC and the MMS2/UBE2N/Ub complex (PDB code; 2GMI). UBE2N and Ub in the UBE2N-Ub product are colored as in Fig. 1a, and MMS2, UBE2N and Ub in the MMS2/UBE2N/Ub complex are colored grey, orange and yellow, respectively. The covalent linkage UBE2N^{K92}-Ub^{Q40} in the UBE2N-Ub product and UBE2N^{C87}-Ub^{G76} are shown in sticks.

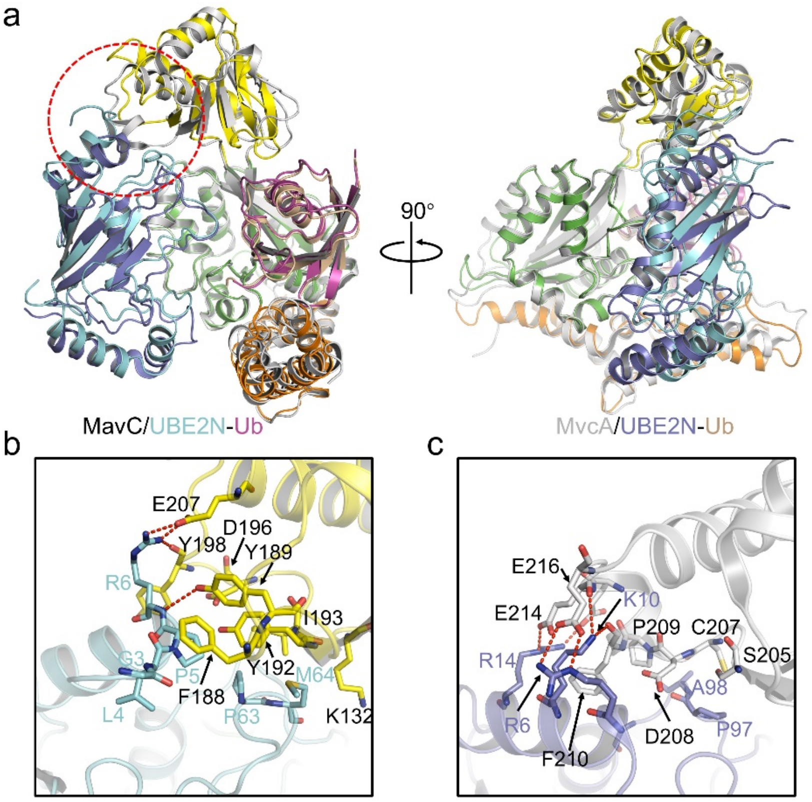

Supplementary Fig. 16 Structural comparisons between MavC/UBE2N-Ub and MvcA/UBE2N-Ub

a. Structural superimposition between the complex of MavC/UBE2N-Ub and MvcA/UBE2N-Ub (PDB: 6JKY). The MavC/UBE2N-Ub is colored as in Fig. 1a. MvcA, UBE2N and Ub in the MvcA/UBE2N-Ub complex are colored grey, slate and wheat, respectively. The MavC/MvcA-UBE2N interface involving the insertion domain is marked in a circle.

b-c. Enlarged view of the region marked by the circle in a. The residues involved in interaction are shown as sticks. Hydrogen bonds are represented as dashed lines.

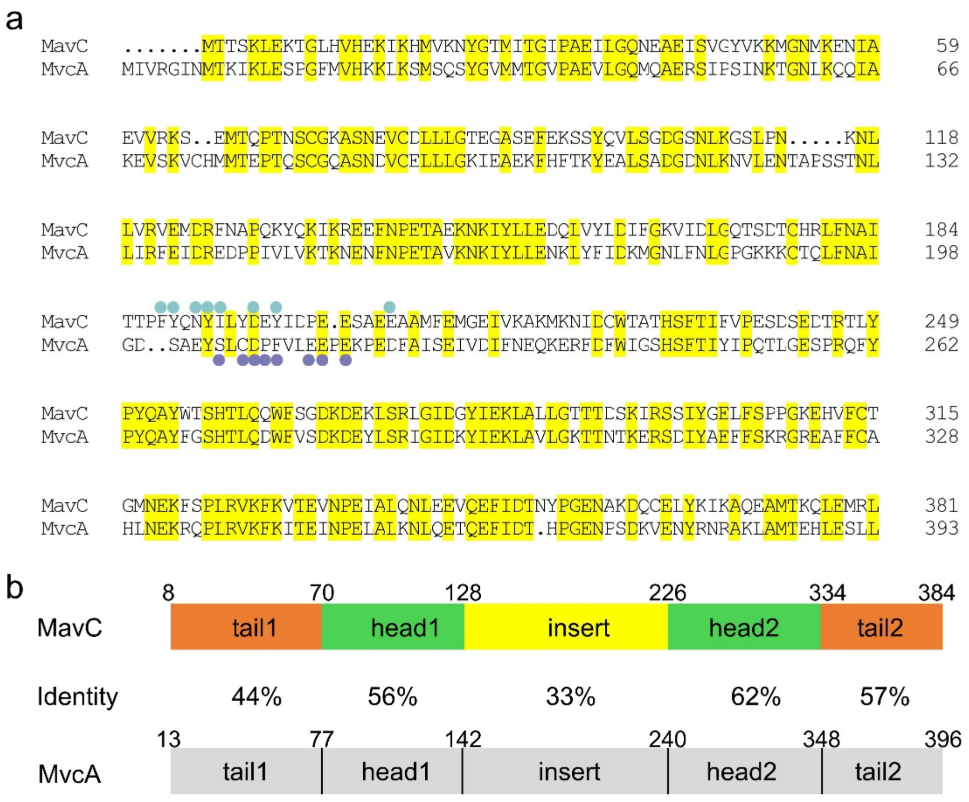

Supplementary Fig. 17 Sequence alignment between MavC and MvcA

a. Sequence alignment between MavC and MvcA with identical residues colored by yellow. The UBE2N-interacting residues within the insertion domains of MavC and MvcA are marked with cyan and slate balls, respectively.

b. Domain architecture of MavC and MvcA. The residue identities between each domain are indicated.

Supplementary Table 1 Primers used in this study

Ub-Nde1f	CCGTCATATGCAGATCTTCGTGAA
Ub-Xho76rstop	CCGCTCGAGTCACCCACCTCGGAGAC
Ub-Q31A-f	ATGTCAAGGCAAAGATCGCAGACAAGGAAGGCATCCC
Ub-Q31A-r	GGGATGCCTTCCTTGTCTGCGATCTTTGCCTTGACAT
Ub-D39A-f	AGGAAGGCATCCCTCCTGCCCAGCAGAGGTTGATCTT
Ub-D39A-r	AAGATCAACCTCTGCTGGGCAGGAGGGATGCCTTCCT
Ub-R72Anew-Xho76rstop	CCGCTCGAGTCACCCACCTCGGAGAGCGAGGACCAGGTGCAGGGT
MavC-BamH1f	CGGGATCCACAACTTCCAAGCTTGA
MavC-Xho482rstop	CCGCTCGAGTTACTTATCACGAAGAACTA
UBE2N-Nde1f	ATACATATGGCCGGGCTGCCCCGCAGGATCAT
UBE2N-Xho152rstop	CCGCTCGAGTTAAATATTATTCATGGCATATAGCCT
MavC-BamH7f	CGGGATCCGAAAAAACGGGTTTGCA
MavC-Xho384rstop	CCGCTCGAGTTACTCTATTAGAAGTCGCA
MavC-C74A-f	CTCAACCCACAAACAGCGCCGGAAAAGCGAGCAATGA
MavC-C74A-r	TCATTGCTCGCTTTTCCGGCGCTGTTTGTGGGTTGAG
UBE2N-Nde1f(P5K)	ATGGCCGGGCTGAAACGCAGGATC
UBE2N-R6E-f	GGGCCCCATATGGCCGGGCTGCCCGAAAGGATCAT
UBE2N-P5GR6E-f	GGGCCCCATATGGCCGGGCTGGGCGAAAGGATCAT
UBE2N-K92A-f	TATGTTTAGATATTTTGGCAGATAAGTGGTCCCCAGC
UBE2N-K92A-r	GCTGGGGACCACTTATCTGCCAAAATATCTAAACATA
E2-E127K-f	TTAGCAAATGATGTAGCGAAACAGTGGAAGACCAACGAA
E2-E127K-r	TTCGTTGGTCTTCCACTGTTTCGCTACATCATTTGCTAA
MavC-F188A-f	ATGCTATTACCACCCCTGCTTATCAAAATTATATTCT
MavC-F188A-r	AGAATATAATTTTGATAAGCAGGGGTGGTAATAGCAT
MavC-Y192A,I193E-f	CCTTTTTATCAAAATGCTGAACTTTATGATGAGTAT
MavC-Y192A,I193E-r	ATACTCATCATAAAGTTCAGCATTTTGATAAAAAGG
MavC-Y192AI193A-f	CCCTTTTTATCAAAATGCTGCTCTTTATGATGAGTATAT
MavC-Y192AI193A-r	ATATACTCATCATAAAGAGCAGCATTTTGATAAAAAGGG
MavC-E203E207A-f	ATTGATCCGGAAGCAAGTGCTGAGGCAGCGGCCATGTTT

MavC-E203E207A-r	AAACATGGCCGCTGCCTCAGCACTTGCTTCCGGATCAAT
MavC-M317A-f	ATGTTTTTTGTACCGGGGCGAACGAAAAATTCTCGCC
MavC-M317A-r	GGCGAGAATTTTTCGTTCGCCCCGGTACAAAAAACAT
MavC-F321A-f	CCGGGATGAACGAAAAAGCCTCGCCATTACGAGTCAA
MavC-F321A-r	TTGACTCGTAATGGCGAGGCTTTTTCGTTCATCCCGG
lpg2149-Nde11f	ATACATATGTTTTTTAAAGATTACCA
lpg2149-Xho114rstop	CCGCTCGAGTTATTTTTGATGAGTAAAGA
lpg2159F11AF12A-Nde11f	ATACATATGGCTGCTAAAGATTACCA
lpg2159F11KF12D-Nde11f	ATACATATGAAAGATAAAGATTACCA
lpg2149-V20L23L24A-BamH1f	CGGGATCCTTTTTTAAAGATTACCAGAAAAAAAATGCAATGAGAGCGGCACAAGACT
lpg2149-Y15K18N19A-BamH1f	CGGGATCCTTTTTTAAAGATGCCCAGAAAGCAGCTGTAATGAG
MavC-BamH128f	CGGGATCCAATGCTCCTCAAAAAT
MavC-Xho226rstop	CCGCTCGAGTTAACAATCTATGTTTTTCA
MavC-127r	TCCAGAACCAAATCGATCCATTTCA
MavC-227f	CGATTTGGTTCTGGATGGACAGCAACCCATTC
MavC-R126A-f	TCAGGGTTGAAATGGATGCATTTAATGCTCCTCAAAA
MavC-R126A-r	TTTTGAGGAGCATTAAATGCATCCATTTCAACCCTGA
MavC-E123A-f	ATTTACTGGTCAGGGTTGCAATGGATCGATTTAATGC
MavC-E123A-r	GCATTAAATCGATCCATTGCAACCCTGACCAGTAAAT
MavC-E123A/R126A-f	CTGGTCAGGGTTGCAATGGATGCATTTAATGCTCCT
MavC-E123A/R126A-r	AGGAGCATTAAATGCATCCATTGCAACCCTGACCAG
NEDD8-Nde1f	GGAATTCCATATGCTAATTAAAGTGAAGACGCTGACCGGA
NEDD8-Xho76rstop	CCGCTCGAGTTATCCTCCTCTCAGAGCCAACACCAGGTGAAG
NEDD8_A72R-Xho76stop	CCGCTCGAGTTATCCTCCTCTCAGACGCAACACCAGGTG
NEDD8-Q39D-f	AAGAGGGAATCCCCCCAGACCAGCAGAGGCTCATCTA
NEDD8-Q39D-r	TAGATGAGCCTCTGCTGGTCTGGGGGGATTCCCTCTT
NEDD8-E31Q-f	ATCAAGGAGCGTGTGCAGGAGAAAGAGGGAATCCC
NEDD8-E31Q-r	GGGATTCCCTCTTTCTCCTGCACACGCTCCTTGAT
NEDD8-K22T-f	CATTGAACCTACAGACACGGTGGAGCGAATCAAGGA

NEDD8-K22T-r	TCCTTGATTCGCTCCACCGTGTCTGTAGGTTCAATG
lpg2149-D84A-f	GATCCACATAAAGCGCTTGCTGCCATGGACAAAAACAT
lpg2149-D84A-r	ATGTTTTTGTCCATGGCAGCAAGCGCTTTATGTGGATC
2149-F71AR74A-r	TGGATCCAGCCAGAGAGCCGTAACAGCATCAGGAA
2149-F71AR74A-f	TTCCTGATGCTGTTACGGCTCTCTGGCTGGATCCA
MavC-31/35/39A-f	ACCGGTGCTCCTGCCGAAGCATTAGGGCAAGCTGAGGCA
MavC-31/35/39A-r	TGCCTCAGCTTGCCCTAATGCTTCGGCAGGAGCACCGGT
MavC-K76E/D83K-f	CTGCGGAGAAGCGAGCAATGAAGTGTGTAAATTACTTT
MavC-K76E/D83K-r	AAAGTAATTTACACACTTCATTGCTCGCTTCTCCGCAG
MavC-E344A/F348A-f	CAAAATTTAGAAGCAGTTCAAGAGGCTATTGATACGAAT
MavC-E344A/F348A-r	ATTCGTATCAATAGCCTCTTGAACTGCTTCTAAATTTTG
MavC-Forward	CGGGATCCATGACAACTTCCAAGCTTG
MavC-Reverse	CGGTCGACTCACTTATCACGAAGAAC
MavC-M317A-Forward	AAGAACATGTTTTTTGTACCGGGGCGAACGAAAAATTCTCGCCATTAC
MavC-M317A-Reverse	GTAATGGCGAGAATTTTTCGTTCGCCCCGGTACAAAAAACATGTTCTT
