# Supplementary material for: Structural insights into the mechanism and inhibition of transglutaminase-induced ubiquitination by the Legionella effector MavC
Source: Nat Commun. 2020 Apr 14;11:1774. doi: 10.1038/s41467-020-15645-7 (PMC7156659; doi:10.1038/s41467-020-15645-7)
Supplement: Supplementary file 3 — Reporting summary [file 41467_2020_15645_MOESM3_ESM.pdf]

nature research

Corresponding author(s): Yue Feng

Last updated by author(s): Mar 16, 2020

Reporting Summary

Nature Research wishes to improve the reproducibility of the work that we publish. This form provides structure for consistency and transparency in reporting. For further information on Nature Research policies, see Authors & Referees and the Editorial Policy Checklist .

Statistics

For all statistical analyses, confirm that the following items are present in the figure legend, table legend, main text, or Methods section.

n/a	Confirmed	
☐	☒	The exact sample size (*n*) for each experimental group/condition, given as a discrete number and unit of measurement
☐	☒	A statement on whether measurements were taken from distinct samples or whether the same sample was measured repeatedly
☒	☐	The statistical test(s) used AND whether they are one- or two-sided<br>*Only common tests should be described solely by name; describe more complex techniques in the Methods section.*
☒	☐	A description of all covariates tested
☒	☐	A description of any assumptions or corrections, such as tests of normality and adjustment for multiple comparisons
☒	☐	A full description of the statistical parameters including central tendency (e.g. means) or other basic estimates (e.g. regression coefficient) AND variation (e.g. standard deviation) or associated estimates of uncertainty (e.g. confidence intervals)
☒	☐	For null hypothesis testing, the test statistic (e.g. *F*, *t*, *r*) with confidence intervals, effect sizes, degrees of freedom and *P* value noted<br>*Give P values as exact values whenever suitable.*
☒	☐	For Bayesian analysis, information on the choice of priors and Markov chain Monte Carlo settings
☒	☐	For hierarchical and complex designs, identification of the appropriate level for tests and full reporting of outcomes
☒	☐	Estimates of effect sizes (e.g. Cohen's *d*, Pearson's *r*), indicating how they were calculated

Our web collection on statistics for biologists contains articles on many of the points above.

Software and code

Policy information about availability of computer code

Data collection	HKL2000 (version 716)
Data analysis	CCP4i 7.0.078, Graphpad Prism 8.0.1, PyMOL 1.8.0.0 , ImageJ 1.52a, COOT 0.8.9.1, PHENIX 1.17.1-3660, Origin 8.0, ASTRA 6.1

For manuscripts utilizing custom algorithms or software that are central to the research but not yet described in published literature, software must be made available to editors/reviewers. We strongly encourage code deposition in a community repository (e.g. GitHub). See the Nature Research guidelines for submitting code & software for further information.

Data

Policy information about availability of data

All manuscripts must include a data availability statement. This statement should provide the following information, where applicable:

- Accession codes, unique identifiers, or web links for publicly available datasets
- A list of figures that have associated raw data
- A description of any restrictions on data availability

Coordinates and structure factors for the complexes have been deposited in the Protein Data Bank (PDB) under accessions: 6KFP, MavC/UBE2N/Ub; 6KG6, MavC/UBE2N-Ub; and 6K3B, MavC/Lpg2149. MavC (PDB: 5TSC), UBE2N (PDB: 1J7D), Lpg2149 (PDB: 5DPO), NEDD8 (1NDD) and Ub (PDB: 1UBQ) were referenced in the manuscript. The source data underlying Figs 1i, 2e–i, 3e, 4e, 5a-b and d-e and Supplementary Figs 4a-b, 6c-d, 7a-c, 8a-g, 11b-f and 12a-b are provided as a Source Data file. Other data are available from the corresponding author upon reasonable request.

Field-specific reporting

Please select the one below that is the best fit for your research. If you are not sure, read the appropriate sections before making your selection.

Life sciences study design

All studies must disclose on these points even when the disclosure is negative.

Sample size	No sample size calculation was performed. For in vitro biochemical studies, three independent experiments were widely accepted and used in published papers.
Data exclusions	No data were excluded from the analyses.
Replication	The experiments were performed independently for at least three times.
Randomization	Randomization is not relevant to the majority of experiments of this study, because protein samples are not required to be allocated into experimental groups in the in vitro activity assays and biochemical studies, and no animals or human research participants are involved in this study. Randomization was used only in structure refinement (Rfree) and it was random.
Blinding	Not applicable. No animals or human research participants are involved in this study, blinding was not used in in vitro studies.

Reporting for specific materials, systems and methods

We require information from authors about some types of materials, experimental systems and methods used in many studies. Here, indicate whether each material, system or method listed is relevant to your study. If you are not sure if a list item applies to your research, read the appropriate section before selecting a response.

Materials & experimental systems

n/a	Involved in the study	
☐	☒	Antibodies
☐	☒	Eukaryotic cell lines
☒	☐	Palaeontology
☒	☐	Animals and other organisms
☒	☐	Human research participants
☒	☐	Clinical data

Methods

n/a	Involved in the study	
☒	☐	ChIP-seq
☒	☐	Flow cytometry
☒	☐	MRI-based neuroimaging

Antibodies

Antibodies used	anti-UBE2N (Thermo Fisher Scientific cat. no. 37-1100), 1:1,000; anti-MavC (Gan, N. et al. Nat Microbiol. 4, 134-143, 2019), 1:5000; anti-tubulin (DSHB, E7) 1:10,000; ICDH (Xu, L. et al. Plos Pathog. 6, e1000822, 2010), 1:10,000.
Validation	Anti-UBE2N (Thermo Fisher Scientific cat. no. 37-1100) has species reactivity for Human, Mouse and Rat, this antibody could be used for ELISA, Immunofluorescence (IF), Western Blot (WB) and Immunoprecipitation (IP); Anti-tubulin (DSHB, E7) has species reactivity for Chlamydomonas, Drosophila, Flatworm, Giant panda, Human, Kangaroo Rat, Mouse, Xenopus, this antibody could be used for Immunohistochemistry (IHC), IF, WB and IP. Purified His6-MavC and His-ICDH have been used to raise rabbit specific antibodies using a standard protocol (Pocono Rabbit Farm & Laboratory), those two antibodies have species reactivity for Legionella and could be applied for WB, IF and IP.

Eukaryotic cell lines

Policy information about cell lines

Cell line source(s)	Raw264.7 cells and U937 cells were purchased from ATCC.
Authentication	All cell lines were authenticated by ATCC through short tandem repeat (STR) profiling.
Mycoplasma contamination	All cell lines tested negative for mycoplasma contamination.
Commonly misidentified lines (See ICLAC register)	No commonly misidentified cell lines were used in the study.
